# The Effectiveness of Dichloroacetate on Human Glioblastoma Xenograft Growth Depends on Na+ and Mg2+ Cations

**DOI:** 10.1177/1559325821990166

**Published:** 2021-02-27

**Authors:** Donatas Stakišaitis, Eligija Damanskienė, Rūta Curkūnavičiūtė, Milda Juknevičienė, Marta Maria Alonso, Angelija Valančiūtė, Saulius Ročka, Ingrida Balnytė

**Affiliations:** 1 Department of Histology and Embryology, Medical Academy, Lithuanian University of Health Sciences, Kaunas, Lithuania; 2 Laboratory of Molecular Oncology, National Cancer Institute, Vilnius, Lithuania; 3 Department of Pediatrics, Clínica Universidad de Navarra, University of Navarra, Pamplona, Spain; 4 Centre of Neurosurgery, Clinic of Neurology and Neurosurgery, Faculty of Medicine, Vilnius University, Vilnius, Lithuania

**Keywords:** glioblastoma, dichloroacetate, magnesium, sodium, PCNA, EZH2, chicken embryo chorioallantoic membrane model

## Abstract

The study’s aim was to investigate the effectiveness of sodium dichloroacetate (NaDCA) or magnesium dichloroacetate (MgDCA) on adult U87 MG and pediatric PBT24 cell lines glioblastoma (GB) xenografts in a chicken chorioallantoic membrane (CAM) model. The study groups were: treated with 10 mM, 5 mM of NaDCA, and 5 mM, 2.5 mM of MgDCA, and controls. The U87 MG and PBT24 xenografts growth, frequency of tumor invasion into CAM, CAM thickening, and the number of blood vessels in CAM differed depending on the dichloroacetate salt treatment. NaDCA impact on U87 MG and PBT24 tumor on proliferating cell nunclear antigen (PCNA) and enhancer of zeste homolog 2 (EZH2) expression in the tumor was different, depending on the NaDCA dose. The 5 mM MgDCA impact was more potent and had similar effects on U87 MG and PBT24 tumors, and its impact was also reflected in changes in PCNA and EZH2 expression in tumor cells. The U87 MG and PBT24 tumor response variations to treatment with different NaDCA concentration on tumor growth or a contrast between NaDCA and MgDCA effectiveness may reflect some differences in U87 MG and PBT24 cell biology.

## Introduction

The most widespread brain tumor is glioblastoma (GB); this tumor accounts for 80 % of all primary malignant central nervous system tumors.^1,2^ GB includes primary (*de novo*) and secondary tumors developed from lower-grade astrocytoma or oligodendroglioma subtypes. Primary GB accounts for 80 % of all GB adult patients, and about 3 % among children with all brain tumors.^3^

The impact of therapy on high-grade GB is limited, as the overall outcome remains unaffected, with an adult patient median survival of 12–15 months.^4^ Five-year survival in pediatric GB patients is less than 20 %,^5^ and the survival in patients aged 55–64 is 6 %^6^ The pharmacological effects of medicines with which GB treatment improves patient survival are related to the inhibition of tumor cell proliferation.^7^ The standard therapy for GB in adults is surgical resection, radiotherapy, and chemotherapy with temozolomide (TMZ).^8^ No standard GB chemotherapy approach has been developed for pediatric patients. Following the progress of TMZ in adults, the treatment has similarly exercised the use of TMZ with radiotherapy for GB pediatric patients.^5^ Individual TMZ effectiveness depends on the development of resistance to TMZ, and this would lead to GB recurrence and a worse GB prognosis.^9^

The usage of sodium dichloroacetate (NaDCA) for sensitizing TMZ-resistant cells has been proposed.^10,11^ The pharmacological NaDCA mechanisms include the inhibition of pyruvate dehydrogenase kinases maintaining the mitochondrial oxidation of glucose, and reduction of lactic acid generation,^12,13^ and a rise in reactive oxygen species generation in the tumor and microenvironment.^14^ Glycolytic processes are upregulated,^15-17^ and expression of pyruvate dehydrogenase kinase is increased in glioma cells.^17,18^ NaDCA treatment enhances cancer cell apoptosis.^19^ A recent study reported that NaDCA works as a Na^+^–K^+^–2Cl^−^ cotransporter (NKCC1) inhibitor, and induces NKCC1 RNA expression suppression.^20^ The NKCC1 inhibition reduces the intracellular chloride level ([Cl^−^]i); a [Cl^−^]i could have an impact on cancer cell proliferation and apoptosis.^21,22^

The unequal anticancer efficacy of NaDCA in *in vitro* and *in vivo* studies raises some questions about the effectiveness of the medicine used in the research.^23^ The NaDCA effectiveness problem may arise as some investigators do not consider the Na^+^ concentration state using high NaDCA doses in *in vitro* studies^24^; it is essential to assess whether the NaDCA concentration in the assay medium will not result in a hypernatraemic state. In humans, a level higher than the 150 mM level of Na^+^ in the blood serum represents hypernatraemia.^25^ Furthermore, researchers have shown that sodium and chloride ions in a tumor microenvironment are involved in cancer progression.^26^ Thus, different concentrations of Na^+^ in the environment of cancer cells under investigation may also modify cancerogenesis.

The chicken chorioallantoic membrane (CAM) model is an alternative model for studying cancer treatment approaches for tumor growth, invasion, angiogenesis, and is one which helps elucidate target medicines for human cancer treatment.^27,28^ The formed human cancer cell tumor on CAM reflects the primary tumor malignancy. DeBord *et al*. show that the CAM model is a helpful tool providing a biologically efficient patient-derived xenograft platform to achieve practical personal potential chemotherapy goals.^29^ A CAM model is more successful in predicting patients-derived xenografted tumor treatment prognosis as compared with rodent immunodeficient models.^30^ A study comparing the effect of the drug on U87 MG tumor growth on CAM with data on medicine exposure to U87 MG spheroids showed that the spheroid model did not reflect the tumor progression or treatment effect on the tumor observed in the CAM model.^31^

The present study aimed to examine differences between a 44-year-old woman high-grade U87 MG cell line^32^ xenograft (U87) and a 13-year-old boy high-grade PBT24 cell line^33^ xenograft (PBT24) studied *in vivo* using the CAM model; to examine these tumors’ response to treatment with NaDCA or magnesium dichloroacetate (MgDCA) preparations as well as their effect on proliferating cell nuclear antigen (PCNA) and enhancer of zeste homolog 2 (EZH2) expression in tumor cells.

## Material and Methods

### Cell Lines and Cell Culturing

A Caucasian adult female’s high-grade glioblastoma U87 MG cell line cells, provided by Dr. Arūnas Kazlauskas (Laboratory of Neuro-Oncology and Genetics, Neuroscience Institute, Lithuanian University of Health Sciences, LT-50009 Kaunas, Lithuania) and a pediatric Caucasian 13-year-old boy’s high-grade glioblastoma PBT24 cell line cells, donated by Prof. Marta M. Alonso (University of Navarra, Spain), were studied. The U87 MG cells were cultivated in Dulbecco’s Modified Eagle Medium (DMEM) medium (Sigma Aldrich, USA), while the PBT24 cells were cultivated in Roswell Park Memorial Institute 1640 (RPMI-1640) medium (Sigma Aldrich, USA). The media were supplemented with a 10 % fetal bovine serum (FBS; Sigma Aldrich, USA) containing 100 IU/mL of penicillin and 100 µg/mL of streptomycin (P/S; Sigma Aldrich, USA). Cells were incubated at 37 °C in a humidified 5 % CO_2_ atmosphere.

### The CAM Model

According to the legislation in force in the EU and Lithuania, no approval for studies using the CAM model is needed from the Ethics Committee. Cobb 500 fertilized chicken eggs were obtained from a local hatchery (Rumšiškės, Lithuania), kept in the incubator (Maino incubators, Italy) at 37 °C temperature and 60 % relative air humidity. The eggs were rolled automatically once per hour until the third embryo development day (EDD3).

The CAM was detached from the shell of the egg at EDD3; eggshells were cleaned with 70 % ethanol, a small round hole was drilled in the location of the air chamber, and approximately 2 ml of the egg white was aspirated. A window of about 1 cm^2^ in the eggshell was drilled and sealed with sterile transparent plastic tape. The eggs were kept in the incubator without rotation until GB cell tumor grafting on CAM at the EDD7.

### The Tumor Study Design

#### Justification for selection of the NaDCA and MgDCA dose

The molecular formula of NaDCA [Cl_2_CHCOONa] indicates that in the solution of 10 mM of NaDCA, there is 10 mM of the 2,2-dichloroacetate anion (DCA) and in the solution of 5 mM of MgDCA [(Cl_2_CHCOO)_2_Mg], there is also 10 mM of the DCA.^34,35^ Respectively, 5 mM concentrations of active substance will be achieved with 5 mM NaDCA and 2.5 mM MgDCA solutions. The concentration of 10 mM NaDCA will not form a hypernatremic state in the used media; the Na^+^ concentration in DMEM medium is 146.4 mM/l and for the RPMI-1640 it is 147.98 mM/l. Other investigators have used a 10 mM of NaDCA dose for cancer cell studies *in vitro.* ^36,37^

#### The U87 and PBT24 tumor study groups

The growth and invasion into CAM of formatted U87 MG cell, as well as of PBT24 cell lines xenografts, were investigated in the 10 groups. The study groups were as follows: the U87-control (the non-treated group, n = 20), the U87 tumors treated with 10 mM of NaDCA (U87-10 mM NaDCA; n = 24), U87-5 mM NaDCA (n = 25), U87-5 mM MgDCA (n = 14), and U87-2.5 mM MgDCA (n = 11). The studied PBT24 tumor groups were the following: PBT24-control (n = 13), PBT24-10 mM NaDCA (n = 12), PBT24-5 mM NaDCA (n = 13), PBT24-5 mM MgDCA group (n = 11), and PBT24-2.5 mM MgDCA (n = 11). When U87 tumors were found to be more sensitive to 5 mM NaDCA than to 10 mM NaDCA, larger groups were tested accordingly to ensure that this was not a random phenomenon.

Biomicroscopy *in vivo* and histological analysis of invasion, the thickness of CAM, and the number of vessels in CAM under the tumor were performed.

The immunohistochemical (IHC) expression of PCNA in the tumor was studied in the following groups: U87-control (n = 8), U87-10 mM NaDCA (n = 8), U87-5 mM NaDCA (n = 8), U87-5 mM MgDCA (n = 8), U87-2.5 mM MgDCA (n = 8); PBT24-control (n = 9), PBT24-10 mM NaDCA (n = 8), PBT24-5 mM NaDCA (n = 6), PBT24-5 mM MgDCA (n = 6), and PBT24-2.5 mM MgDCA (n = 7).

The expression of EZH2 was investigated in the following: U87 tumors control (n = 12), U87-10 mM NaDCA (n = 13), U87-5 mM NaDCA (n = 12), U87-5 mM MgDCA (n = 7), and U87-2.5 mM MgDCA (n = 8); PBT24-control (n = 6), PBT24-10 mM NaDCA (n = 7), PBT24-5 mM NaDCA (n = 6), PBT24-5 mM MgDCA (n = 7), and PBT24-2.5 mM MgDCA (n = 7).

### Biomicroscopy Data to Assess Tumor Growth and Drug Efficacy

The biomicroscopy of xenografts on CAM at embryo development from 9 to 12 days (EDD9–12) *in vivo* is suitable to evaluate the tumor growth characteristics, its malignancy, and to detect the disparities among different cell line tumors and the sensitivity to treatment. One kind of tumor malignancy and growth progression signs is a relatively rapid formation of vasculature around the tumor—a “spoked-wheel” consisting of tumor-attracted small blood vessels, and formed by neoangiogenesis new blood vessels. The tumor size, its border clarity, and changes of the “spoked-wheel” expression may serve as features of the drug effect on tumorigenesis.

### Tumor Grafting on CAM In Vivo

An absorbable gelatin surgical sponge (Surgispon, Aegis Lifesciences, India) was cut manually with a blade into pieces of 9 mm^3^ (3 × 3 × 1 mm). The 1 × 10^6^ cells were resuspended in 20 µl of the rat tail collagen, type I (Gibco, USA) (in the control group), and MgDCA or NaDCA salt (Sigma-Aldrich GmbH, Germany) dissolved in a medium (investigational medicine-treated groups). A 20 µl liquid mixture of tumor cells was pipetted onto a piece of a sponge. The NaDCA- or MgDCA-treated tumor groups and their controls were formed. At the EDD7, the tumor was grafted onto CAM among significant blood vessels. Its structural changes were observed with a stereomicroscope (SZX2-RFA16, Olympus, Japan) *in vivo* during the EDD9–12 period. The tumor images were acquired using a digital camera (DP92, Olympus, Japan) and CellSens Dimension 1.9 Digital Imaging Software.

### Histological Study of the Tumor

At EDD12, the specimens were harvested, fixed in a buffered 10 % formalin solution for 24 hours and embedded into the paraffin wax. The tumor sample was cut with a microtome (Leica, Germany) into 3 µm thickness sections. The sections were stained with hematoxylin and eosin (H–E) and by IHC methods. Visualization and photographing of H–E and IHC stained tumor slides were performed using a light microscope (BX40F4, Olympus, Japan) and a digital camera (XC30, Olympus, Japan) equipped with CellSens Dimension 1.9 software.

H–E stained tumors were divided into 2 types: invasive and non-invasive. The tumor invasion into CAM was categorized as the destruction of the chorionic epithelium (ChE) or/and tumor cell invasion into the CAM mesenchyme. The tumor not invaded into mesenchyme was located on the surface of the CAM, and the integrity of the chorionic epithelium was not disrupted. The tumor invasion was examined in H–E slides at 20 × and 40 × magnifications.

### Assessment of the CAM Thickness and the Number of Blood Vessels in CAM

The CAM thickness (width) was evaluated by photographing H–E stained CAM at 4 × magnification directly under the tumor. The thickness of CAM was measured (µm) in 10 areas. The median CAM thickness was calculated in the area under the tumor. In the CAM without tumor group, 3 random places of CAM were selected and measured accordingly.

The number of blood vessels was assessed by photographing the H–E stained CAM at 4 × magnification directly under the tumor. In the CAM without tumor group, one random vision field was selected. Blood vessels bigger than 10 µm were counted in the same length of the CAM (1792 µm).

### Immunohistochemical Study

The expression of the PCNA and EZH2 markers was determined in tumor cells by immunohistochemistry. Primary antibodies to the PCNA (PC10, Thermo Fisher Scientific, USA) and the KMT6/EZH2 (phospho S21, ab84989, Abcam, UK) were used to detect PCNA- and EZH2-positively stained tumor cells. Thin CAM sections of 3 µm were mounted onto adhesion slides (Thermo Fisher Scientific, USA), deparaffinized, and rehydrated by standard techniques. The heat-induced antigen retrieval was performed using a Tris/EDTA buffer at pH 9 (K8002, Dako, Denmark) and a pressure cooker at 95 °C for 20 minutes (Thermo Fisher Scientific, USA). The Shandon CoverPlate System (Thermo Fisher Scientific, USA) was used for staining. Endogenous peroxidase was blocked with the Peroxidase Blocking Reagent (SM801, Dako, Denmark). The slides were treated with primary antibodies (1:100) for 30 minutes at room temperature. The primary antibody and antigen complex was determined using the horseradish peroxidase-labeled polymer dextran conjugated with a secondary mouse antibody and a linker (SM802 and SM804, respectively; Dako, Denmark) for 30 min. at room temperature. Positive reactions were visualized using the 3,3’-diaminobenzidine-containing chromogen (DAB, DM827, Dako, Denmark), which gives the brown color to the site of the target antigen recognized by the primary antibody. After each step, a tris-buffered saline solution containing Tween 20 (DM831, Dako, Denmark) was used as a wash buffer. Slides were counterstained with the Mayer hematoxylin solution (Sigma Aldrich, Germany), dehydrated, cleared, and mounted.

For assessment of the tumor PCNA and EZH2 protein expression, 2 random vision fields (plot area 23863.74 µm^2^) of the immunohistochemically stained tumor were photographed at 40 × magnification. All cells, and the PCNA-, EZH2-positively stained cells were calculated in selected vision fields, and the percentages of PCNA-and EZH2-positive cells were counted in each tumor.

### Statistical Analysis

The statistical analysis was performed using the Statistical Package for Social Sciences, version 23.0 for Windows (IBM SPSS Statistics V23.0). The frequency of invasion into CAM is expressed as a percentage (%), and the chi-square test was used to compare tumor invasion into CAM frequency between the study groups. The Shapiro-Wilk test was used to verify the normality assumption. Data of PCNA- and EZH2-positive stained cells, the number of blood vessels, and the thickness of the CAM are expressed as median and range (minimum and maximum values). The difference between the 2 independent groups was evaluated using the nonparametric Mann-Whitney U test. The Spearman rank correlation coefficient (r) was used to assess the relationship between the CAM thickness and the number of blood vessels. Differences at the value of *P* < 0.05 were considered significant.

## Results

### The Biomicroscopy of U87 Xenograft on CAM

**Figure 1.**
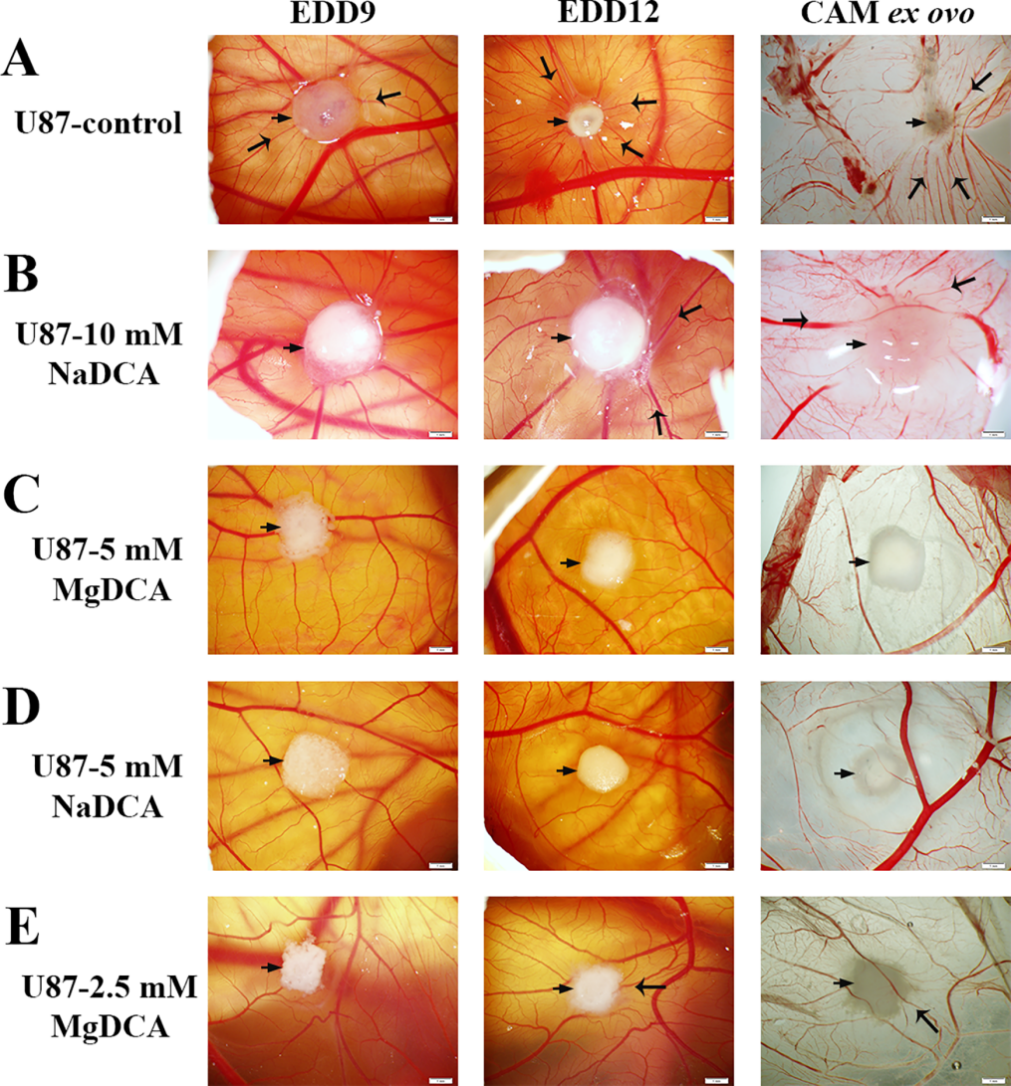
Biomicroscopy of U87 tumor on CAM *in vivo* and the chorioallantoic membrane with tumor *ex ovo* of the study groups (A, B, C, D, E). The EDD9 pictures represent the tumor 2 days after the xenograft transplantation, and EDD12—after 5 days of transplantation on CAM. The *ex ovo* photos represent the harvested CAM with a tumor on EDD12. A short arrow shows the tumor, a longer arrow—the vessel of the “spoked-wheel” in the U87-control (A, EDD12, and CAM *ex ovo*); it is a reduced expression in the treated tumor (B, E, EDD12, and CAM *ex ovo*) or suppressed the development of vascular network (C, D, EDD12, and CAM *ex ovo*). Scale bar—1 mm.

Figure 1 shows the biomicroscopy data dynamics of U87-control and tumor treated with 10 mM, 5 mM of NaDCA, and with 5 mM or 2.5 mM of MgDCA doses, from EDD9 to EDD12, and the harvested CAM with tumor at EDD12. The U87-control tumor has distinct edges at EDD9; its size diminishes at EDD12; the smaller size of the non-treated tumor at EDD12 is associated with its invasion into CAM mesenchyme (Figure 1-A), the vessels plexus is weakly expressed at EDD9; a dense vessels network is formed in the direction of the tumor at EDD12: a manifested “spoked-wheel” pattern is seen 5 days after tumor grafting on CAM. The apparent network of vessels around the non-treated tumor is displayed in harvested CAM with a tumor at EDD12 (Figure 1-A).

Figure 1-B shows U87-10 mM NaDCA tumor growth during EDD9–12; the tumor visually appears to be larger *in vivo* and *ex ovo* compared with the U87-control at EDD9 and EDD12 because the tumor grows more on the CAM surface; a “spoked-wheel” at EDD12 is less expressed as compared with the control. The effect of 10 mM dichloroacetate anion of different salts on U87 tumor (Figure 1-B, and C) was different; it can be seen that MgDCA salt inhibits tumor growth significantly more: both tumors grow on the CAM surface, but treated with 5 mM MgDCA, the tumor is smaller with no visible vascular network around the tumor. U87 tumor growth was more strongly affected by 5 mM of NaDCA than by 10 mM of NaDCA (Figure 1-B, and D). The ‘spoked-wheel” development around the U87-2.5 mM MgDCA tumor (Figure 1-E, EDD12, and CAM *ex ovo*) indicates that tumor growth suppression is limited.

### The Biomicroscopy of PBT24 Xenograft on CAM

**Figure 2.**
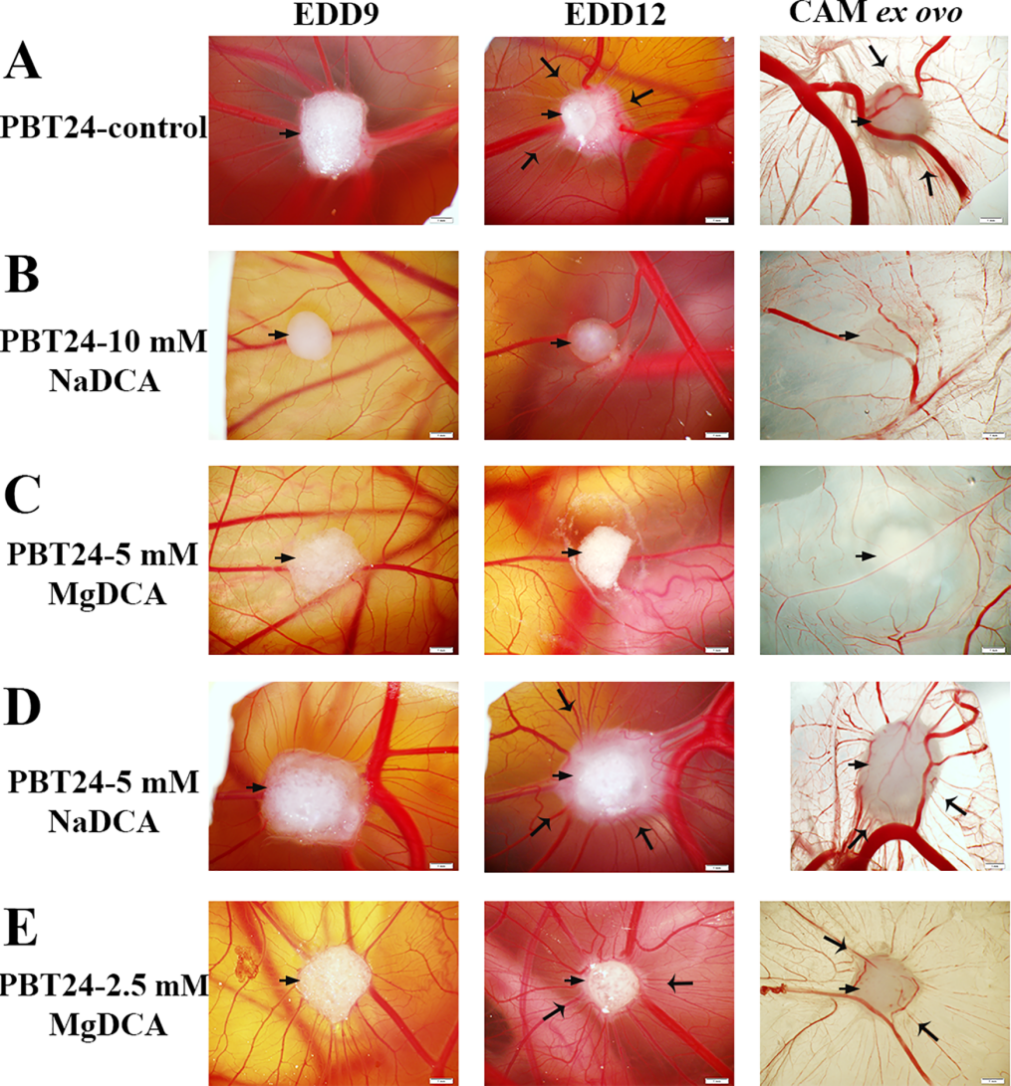
Biomicroscopy of PBT24 tumor in vivo and chorioallantoic membrane with tumor ex ovo of the study groups (A, B, C, D, E). The EDD9 pictures show the tumor 2 days after, and the EDD12—5 days after its transplantation on CAM. Ex ovo pictures represent the harvested tumor with CAM at EDD12. A short arrow shows the tumor, a more extended indicator—a “spoked-wheel” around the tumor at EDD12 and in respectively ex ovo preparations (A, D, and E). Scale bar—1 mm.

Figure 2 shows the data dynamics of PBT24-control and treated tumor studied groups from EDD9 to EDD12 as well as the harvested CAM preparation with a tumor at EDD12. The control xenograft has precise contours on EDD9, but they are less clear on EDD12 and the tumor size is slightly reduced (Figure 2-A) because the tumor has partially invaded into the mesenchyme; its apex is visible on the surface of a chorionic epithelium, and around the tumor is the expressed “spoked-wheel” (Figure 2-A, EDD12, and CAM *ex ovo*).

Compared with the control, the PBT24 treatment with 10 mM of NaDCA and with 5 mM of MgDCA suppresses the tumor growth and vascular network development, with tumor edges visible, indicating tumor growth on the CAM surface (Figure 2-B, and C). The PBT24-5 mM NaDCA tumor growth is poorly inhibited, the tumor grows more on the CAM surface, and the “spoked-wheel” is visible (Figure 2-D). Compared with the control, the xenograft-treated with 2.5 mM of MgDCA was partially invasive (its edges unclear) with only partially inhibited development of the vascular network at EDD12 (Figure 2-E).

### The Biomicroscopically Determined Differences Among U87 and PBT24 Tumors

The data indicate that U87 and PBT24 tumors show different sensitivity to treatment with the sodium salt of dichloroacetate (Figures 1 and 2). A 10 mM dose of NaDCA was less effective in the treatment of the U87 tumor as compared with 5 mM of NaDCA, but a 10 mM dose was effective in treating the PBT24 tumor. The PBT24 tumor was quite insensitive to treatment with 5 mM NaDCA (Figure 2). The 5 mM MgDCA dose (10 mM of dichloroacetate anion) was similarly effective in U87 and PBT24 tumor treatment.

### Histological Tumor Data in Evaluating the Effectiveness of the Treatment

**Figure 3.**
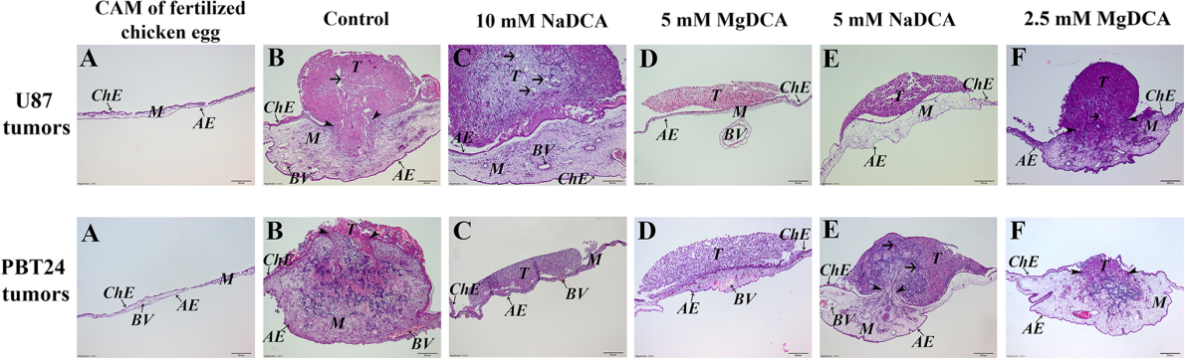
The histologic images of CAM, tumor growth, and its invasion into the CAM frequency in the U87 and PBT24 study groups. A—CAM of a fertilized chicken egg without tumor, B—control tumor, C—10 mM NaDCA, D—5 mM MgDCA, E—5 mM NaDCA tumor, F—2.5 mM MgDCA. Arrowhead indicates the destruction of the chorionic epithelium; shorter arrow shows blood vessels formed around the tumor. ChE—chorionic epithelium, AE—allantoic epithelium, M—mesenchyme, BV—blood vessel, T—tumor. Scale bar—200 µm.

**Figure 4.**
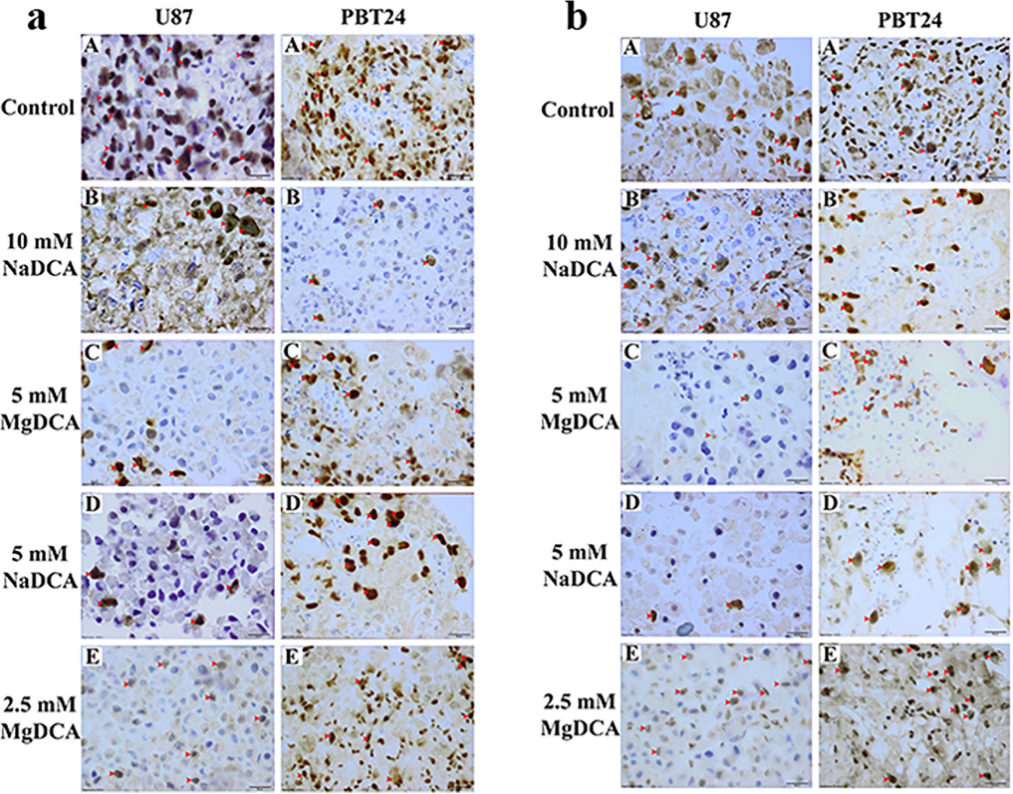
PCNA- and EZH2-positive stained tumor of the U87 and PBT24 study groups. **a**. PCNA-positive stained tumor of the U87 and PBT24 study groups. A—control, B—10 mM NaDCA, C—5 mM MgDCA, D—5 mM NaDCA, and E—2.5 mM MgDCA treated tumors. Red arrowhead indicates positively stained cells. Scale bar—20 µm. **b**. EZH2-stained cells in the U87 and PBT24 study groups. A—control, B—10 mM NaDCA, C—5 mM MgDCA, D—5 mM NaDCA, and E—2.5 mM MgDCA tumor. Red arrowhead indicates EZH2-positive cells. Scale bar—20 µm.

The histological examination provides a more detailed explanation of the response of U87 and PBT24 to the treatment applied, assessing the tumor growth, tumor invasion into CAM mesenchyme frequency, and a thickening of the CAM beneath the xenograft in the study groups (Figure 3). The CAM thickness is related to the activation of neoangiogenesis, assessed by the number of blood vessels in CAM under the tumor (Figure 3; Tables 1 and 2). Immunohistochemical study of the PCNA and EZH2 expression in the xenograft cells at EDD12 enabled the evaluation of the features of tumorigenesis, as well as differences in the treatment effect with dichloroacetate salts among U87 MG and PBT24 cell line tumors (Figure 4; Tables 3 **and** 4 **).**

**Table 1.** The U87 Tumor Invasion Into CAM Frequency, the Thickness of CAM and the Number of a Blood Vessel in CAM Under the Tumor of the Study Groups.

Study group	n	Invasion (%)	CAM thickness (μm)	Number of blood vessels
			median (range)	median (range)
U87-control	20	80.00	197.80 (65.20−387.94)	23.50 (9−63)
U87-10 mM NaDCA	24	45.83^a^	219.20 (65.41−1139.45)^g^	22.0 (4−61)
U87-5 mM MgDCA	14	14.29^b,c^	105.16 (35.87–579.59)^h^	18.0 (6–35)^j^
U87-5 mM NaDCA	25	20.00^d,e^	149.20 (49.99−452.53)^i^	10.0 (2−40)^k^
U87-2.5 mM MgDCA	11	36.36^f^	142.51 (49.44−378.32)	20.0 (6−32)^l^

^a^ *P* = 0.02, compared with U87-control.

^b^ *P* = 0.0008, compared with U87-control.

^c^ *P* = 0.048, compared with U87-10 mM NaDCA.

^d^ *P* < 0.0001, compared with U87-control.

^e^ *P* = 0.044, compared with U87-10 mM NaDCA.

^f^ *P* = 0.015, compared with U87-control.

^g^ *P* = 0.02, compared with U87-5 mM MgDCA.

^h^ *P* = 0.047, compared with U87-control.

^i^ *P* = 0.02, compared with U87-10 mM NaDCA.

^j^ *P* = 0.009, compared with U87-control.

^k^ *P* = 0.0006, compared with U87-control.

^l^ *P* = 0.036, compared with U87-control.

CAM: chorioalantoic membrane; NaDCA: sodium dichloroacetate; MgDCA: magnesium dichloroacetate.

**Table 2.** The Frequency of PBT24 Tumor Invasion Into CAM, the CAM Thickness, and Blood Vessel Number in CAM Under the Tumor in the Study Groups.

Study group	N	Invasion (%)	CAM thickness (µm)	Number of blood vessels
			median (range)	median (range)
PBT24-control	13	76.92	300.88 (65.23–700.87)	15 (6–28)
PBT24-10 mM NaDCA	12	16.67^a^	199.23 (54.44–627.55)	11.5 (3–25)
PBT24-5 mM MgDCA	11	18.18^b^	96.53 (36.28–591.91)^e^	7 (2–27)^g^
PBT24-5 mM NaDCA	13	53.85^c,d^	236.02 (31.27–484.44)	12 (5–29)
PBT24-2.5 mM MgDCA	11	45.45	138.60 (21.34–384.022)^f^	9 (2–23)

^a^ *P* = 0.003, compared with PBT24-control.

^b^ *P* = 0.004, compared with PBT24- control.

^c^ *P* = 0.04, compared with PBT24-10 mM NaDCA.

^d^ *P* = 0.049, compared with PBT24-5 mM MgDCA.

^e^ *P* = 0.013, compared with PBT24-contol.

^f^ *P* = 0.041, compared with PBT24-contol.

^g^ *P* = 0.018, compared with PBT24-control.

CAM: chorioalantoic membrane; NaDCA: sodium dichloroacetate; MgDCA: magnesium dichloroacetate.

**Table 3.** The Percentage of PCNA- and EZH2-Positive Cells in U87 Tumor of the Study Groups.

Study group	PCNA-positive cells (%)		EZH2-positive cells (%)	
	n	median (range)	n	median (range)
U87-control	8	67.73 (15.44–92.73)	12	69.42 (5.34–94.06)
U87-10 mM NaDCA	8	25.70 (1.55–53.89)^a^	13	26.49 (1.88–74.11)^e^
U87-5 mM MgDCA	8	28.53 (3.12–74.99)^b^	7	3.39 (1.14–57.02)^f,g^
U87-5 mM NaDCA	8	15.25 (7.69–30.97)^c,d^	12	8.40 (2.15–92.17)^h,i^
U87-2.5 mM MgDCA	8	44.18 (17.14–87.98)	8	43.99 (14.59–94.77)

^a^ *P* = 0.04, compared with U87-control.

^b^ *P* = 0.049, compared with U87-control.

^c^ *P* = 0.047, compared with U87-control.

^d^ *P* = 0.01, compared with U87-2.5 mM MgDCA.

^e^ *P* = 0.02, compared with U87-control.

^f^ *P* = 0.04, compared with U87-control.

^g^ *P* = 0.009, compared with U87-5 mM NaDCA.

^h^ *P* = 0.003, compared with U87-control.

^i^ *P* = 0.007, compared with U87-2.5 mM MgDCA.

PCNA: proliferating cell nuclear antigen; EZH2: enhancer of zeste homolog 2; NaDCA: sodium dichloroacetate; MgDCA: magnesium dichloroacetate.

**Table 4.** The Percentage of PCNA- and EZH2-Positive Cells in PBT24 Tumor of the Study Groups.

Study group	PCNA-positive cells (%)		EZH2-positive cells (%)	
	n	median (range)	n	median (range)
PBT24-control	9	63.78 (34.87–80.95)	6	71.00 (42.63–78.70)
PBT24-10 mM NaDCA	8	10.46 (2.38–24.34)^a,b^	7	19.59 (14.89–44.58)^g,h^
PBT24-5 mM MgDCA	6	20.70 (16.40–31.68)^c,d^	7	17.93 (2.78–44.44)^i,j^
PBT24-5 mM NaDCA	6	14.97 (9.99–31.06)^e,f^	6	18.83 (9.84–78.70)^k,l^
PBT24-2.5 mM MgDCA	7	43.12 (19.47–93.54)	7	44.70 (21.51–94.76)

^a^ *P* < 0.0001, compared with PBT24-control.

^b^ *P* = 0.0006, compared with PBT24-2.5 mM MgDCA.

^c^ *P* = 0.0004, compared with PBT24-control.

^d^ *P* = 0.0221, compared with PBT24-2.5 mM MgDCA.

^e^ *P* = 0.0004, compared with PBT24-control.

^f^ *P* = 0.0047, compared with PBT24-2.5 mM MgDCA.

^g^ *P* = 0.002, compared with PBT24-control.

^h^ *P* = 0.0262, compared with PBT24-2.5 mM MgDCA.

^i^ *P* = 0.002, compared with PBT24-control.

^j^ *P* = 0.007, compared with PBT24-2.5 mM MgDCA.

^k^ *P* = 0.0221, compared with PBT24-2.5 mM MgDCA.

^l^ *P* = 0.04, compared with PBT24-control.

PCNA: proliferating cell nuclear antigen; EZH2: enhancer of zeste homolog 2; NaDCA: sodium dichloroacetate; MgDCA: magnesium dichloroacetate.

### The U87 Growth, Invasion Into CAM Frequency, the CAM Thickness and the Number of Blood Vessels in CAM Under the Tumor of the Study Groups

Figure 3 shows the treatment efficacy in the inhibition of tumor growth, and the effect of the research medicine on inhibiting tumor adhesion to CAM (Figure 3, U87 tumors, D, and E). Histomorphometric data show that the U87 tumor invasion into CAM frequency was found to depend on the dichloroacetate anion concentration, as well as on the dichloroacetate salt. Tumor invasion frequency in the study groups was as follows: U87-control was 80.0 %, U87-10 mM NaDCA—45.83 %, U87-5 mM MgDCA—14.29 %, U87-5 mM NaDCA—20.0 %, and U87-2.5 mM MgDCA—36.36 % (-Table 1). Compared with the U87-control, the applied treatment significantly reduced the tumor invasion in all study groups (P < 0.05). Comparing the frequency of invasion into CAM of U87-10 mM NaDCA, the inhibition of tumor invasion was found significantly higher in U87-5 mM MgDCA (P = 0.048) and U87-5 mM NaDCA (P = 0.044) groups (-Table 1). No difference in invasion frequency was found between U87-5 mM NaDCA and U87-5 mM MgDCA groups (P > 0.05).

The CAM thickness of the matched control without a grafted tumor was 42.57 µm with the range being from 35.68 to 51.19 µm (n = 10). In the U87-control group, it was 4.7-times more than the CAM thickness without a grafted tumor (*P* < 0.001). Significantly increased CAM thickness of the U87-10 mM NaDCA was found when compared with U87-5 mM MgDCA (*P* = 0.02). CAM thickness was significantly different between U87-control and U87-5 mM MgDCA (*P* = 0.047), and between the U87-5 mM NaDCA and U87-10 mM NaDCA (*P* = 0.02) groups (Table 1).

The number of blood vessels in the CAM under the tumor did not differ between the U87-control and U87-10 mM NaDCA group (*P* > 0.05), but it was significantly decreased in U87-5 mM NaDCA (*P* = 0.0006), when treated with 5 mM of MgDCA (*P* = 0.009), and when treated with 2.5 mM of MgDCA (*P* = 0.036) groups (Table 1).

The significant correlation (*r*) between CAM thickness and the number of blood vessels in CAM was determined in the following U87 study groups: 0.80 in U87-10 mM NaDCA (*P* < 0.0001), 0.45 in U87-5 mM NaDCA (*P* = 0.02), and no such significant correlation was found in other groups.

### The PBT24 Growth, Its Invasion Into CAM Frequency, the Thickness of CAM and the Number of Blood Vessels in CAM Under the Tumor of the Study Groups

Figure 3 gives images of H–E-stained slides of CAM, and the characteristic changes of the PBT24 tumor’s size, formed blood vessels in the CAM under the tumor, and the CAM thickness changes in the PBT24-control and PBT24-treated groups. The chorionic epithelium under the xenograft is destroyed, and the tumor invasion into the CAM mesenchyme is evident in the PBT24-control and PBT24-5 mM NaDCA (Figure 3, PBT24 tumors, B, and E). The chorionic epithelium is intact in 10 mM NaDCA and 5 mM MgDCA, and the tumor is distributed on the CAM surface (Figure 3, PBT24 tumors, C, and D).

In PBT24-control the incidence of tumor invasion to CAM was 76.92 %, in the PBT24-10 mM NaDCA—16.67 %, PBT24-5 mM MgDCA—18.18 %, PBT24-5 mM NaDCA—53.85 %, and in PBT24-2.5 mM MgDCA—45.45 % (Table 2). Comparing the incidence of tumor invasion into CAM of the PBT24-control with treated groups, significantly suppressed invasion frequency was found in treatment with 10 mM of NaDCA (*P* = 0.003) and 5 mM of MgDCA groups (*P* = 0.004). In the PBT24-10 mM NaDCA, the incidence of invasion was statistically less frequent than in the PBT24-5 mM NaDCA group (*P* = 0.04). Treatment with 5 mM of MgDCA effectively inhibited the invasion more than therapy with 5 mM of NaDCA (*P* = 0.049).

The non-treated PBT24 xenograft induces the thickening of CAM mesenchyme (Table 2). The CAM thickness in the PBT24-control was 7-times more than the width of control without a grafted tumor (*P* < 0.001). Comparing the CAM thickness of PBT24-control with that of treated study groups shows a significant CAM thickness decrease only in the PBT24-5 mM MgDCA group (*P* = 0.013), and in the PBT24-2.5 mM MgDCA group (*P* = 0.041).

The PBT24-control and PBT24-5 mM NaDCA tumors are vascularized; blood vessels are visible (Figure 3, PBT24 tumors, B, and E). PBT24 tumors treated with 10 mM NaDCA and 5 mM MgDCA are not vascularized (Figure 3, PBT24 tumors, C, and D). Compared to the PBT24-control, the number of blood vessels in CAM was significantly reduced only in the PBT24-5 mM MgDCA group (*P* = 0.018).

The significant correlation (*r*) between CAM thickness and the number of blood vessels in CAM of PBT24 study groups was as follows: 0.69 (*P* = 0.01) in the PBT24-control, 0.69 (*P* = 0.01) in PBT24-5 mM NaDCA, 0.74 in PBT24-5 mM MgDCA group (*P* = 0.009), and 0.77 (*P* = 0.005) in PBT24-2.5 mM MgDCA group.

### The PCNA Expression in U87 Tumor

The PCNA protein expression was observed in the nucleus of the GB cell, in the nucleus of chorionic, and in the allantoic epithelium and CAM mesenchyme cells. The PCNA expression in tumor tissue is shown in the images, which represent the mean expression of the marker in the studied group (Figure 4-a). The highest expression of PCNA was found in the U87-control, where PCNA-positive cells percentage comprises 67.73 %. The rate of PCNA-positive cells among treated groups was found to depend on the applied treatment: it comprises 25.70 % in U87-10 mM NaDCA, 28.53 % in U87-5 mM MgDCA, 15.25 % in U87-5 mM NaDCA, and 44.18 % in U87-2.5-MgDCA group (Table 3). Compared with the U87-control, PCNA expression was significantly lower in U87-10 mM NaDCA (*P* = 0.04), U87-5 mM MgDCA (*P* = 0.049), U87-5 mM NaDCA (*P* = 0.047). No significant difference in PCNA expression was found among those treated with 10 mM of NaDCA, 5 mM MgDCA and 5 mM of NaDCA groups (*P* > 0.05). There was a significant difference between U87-5 mM NaDCA and U87-2.5 mM MgDCA groups (*P* = 0.01).

### The EZH2 Expression in U87 Tumor

EZH2 protein is expressed in tumor cell nuclei. The appearance of the marker in the tumor is shown in the images, which corresponds to the mean of the EZH2-positive cells of the study group (Figure 4-b). The highest EZH2 expression was in the U87-control where it comprises 69.42 %; 26.49 % in U87-10 mM NaDCA, 3.39 % in U87-5 mM MgDCA, 8.4 % in U87-5 mM NaDCA, and 43.99 % in U87-2.5 mM MgDCA (Table 3). Compared with the control, EZH2 expression was significantly reduced in all treated groups (*P* < 0.05), with the exception of the U87-2.5 mM MgDCA group (*P* > 0.05). When comparing the treated groups, the difference in marker expression was significant between the U87-5 mM MgDCA and U87-5 mM NaDCA (*P* = 0.009), and between U87-2.5 mM MgDCA and U87-5 mM NaDCA groups (*P* = 0.007).

### The PCNA Expression in PBT24 Tumor

Figure 4-a presents images of the PCNA expression in tumor tissue of the PBT24 studied groups. The percentage of PCNA-positive cells was as follows: 63.78 % in the PBT24-control, 10.46 % in PBT24-10 mM NaDCA, 20.70 % in PBT24-5 mM MgDCA, 14.97 % in PBT24-5 mM NaDCA, and 43.12 % in PBT24-2.5 mM MgDCA group (Table 4). Compared to the PBT24-control, the PCNA expression was significantly reduced in the PBT24-10 mM NaDCA group (*P* < 0.0001), PBT24-5 mM NaDCA (*P* = 0.0004), PBT24-5 mM MgDCA (*P* = 0.0004), and no significant treatment effect was found in the PBT24-2.5 mM MgDCA group (*P* > 0.05). The treatment effect of 5 mM of NaDCA was higher than that of 2.5 mM MgDCA treatment (*P* = 0.0047). The effectiveness of 2.5 mM MgDCA treatment was significantly lower than that of 5 mM MgDCA (*P* = 0.022), and of 10 mM NaDCA (*P* = 0.0006) (Table 4).

### The EZH2 Expression in PBT24 Tumor

The EZH2-positive cells percentage in PBT24-control comprises 71.0 % (Table 4). Compared with the control, a significant diminishing of the EZH2-positive cells when treated with the dichloroacetate preparation groups was determined: in PBT24-10 mM NaDCA 19.59 % (*P* = 0.002), PBT24-5 mM MgDCA 17.93 % (*P* = 0.002), PBT24-5 mM NaDCA 18.83 % (*P* = 0.04), and in PBT24-2.5 mM MgDCA 44.7 % (*P* > 0.05). No significant difference in EZH2 expression was found among those treated with 10 mM of NaDCA, 5 mM MgDCA and 5 mM of NaDCA (*P* > 0.05), but there was a significant difference when comparing the expression in PBT24-2.5 mM MgDCA with PBT24-10 mM NaDCA (*P* = 0.0262) and with PBT24-5 mM NaDCA (*P* = 0.022). The treatment with 5 mM MgDCA significantly reduced EZH2 expression more than treatment with 2.5 mM MgDCA dose (*P* = 0.007) (Table 4).

### Histological Differences Among U87 and PBT24 Tumors

There was no difference in the incidence of tumor invasion into CAM in U87- and PBT24-controls. The treatment of tumors with 5 mM of NaDCA significantly reduced the U87 tumor invasion into CAM but had no apparent effect on the PBT24 tumor (Tables 1 and 2). Significant differences in CAM width as the effect of different doses of NaDCA were found on U87, but no such difference was found in the PBT24-treated groups (Tables 1 and 2). No difference in the number of blood vessels in the U87- and PBT24-control was found, but MgDCA with a dose-dependent effect significantly reduced their number only in the U87 tumor groups. Compared with the corresponding controls, PCNA expression in U87 tumors was mainly reduced by 5 mM NaDCA (4.4-fold) and in PBT24 by 10 mM NaDCA (6.1-fold). The EZH2-positive cell number in the U87 tumor decreased the most with 5 mM NaDCA and 5 mM MgDCA treatment by 8.3- and 20.5-fold, respectively, while in the PBT24 tumor by 3.8- and 4.0-fold, respectively.

## Discussion

Pediatric high-grade GB is less common in the general population when compared to just adults. The genomic data has improved our knowledge and showed that the nature of pediatric GB is distinct from that seen in adults.^33,38^ The search for more effective adult and pediatric GB treatment has met with unsatisfactory progress for decades.^38,39^ GB therapy requires a personalized approach with determining tumor treatment sensitivity in the preclinical stage.^39,40^ The nature of the GB cells or treatment-related effects on cell glycolytic track changes, can be one approach for cancer therapy.^17,18,41^ The monotherapy with NaDCA, as a mitochondrial regulator, illustrates antitumor effects in *in vivo* models.^24^

The study of U87 and PBT24 tumors control peculiarities indicate their similar malignancy. The biomicroscopic, histomorphological analysis revealed that U87 and PBT24 tumors exhibit different sensitivity between treatment with NaDCA and MgDCA; that the efficacy of investigational medicines for xenograft growth, invasion into CAM, angiogenesis depends not only on the dichloroacetate anion concentration but also on the cation in the salt.

NaDCA monotherapy *in vivo* significantly inhibited U87 MG cells subcutaneous tumor growth in the male Balb/c nude mouse model.^42^ Others did not find a monotherapy effect on the U87 MG tumor in mouse brain growth in the athymic nude female mice model,^43^ and in the subcutaneous U87 MG-derived xenograft in the athymic nude female mice.^36^ U87 MG cell spheroids showed resistance to 10 mM of NaDCA treatment.^36^ No studies of NaDCA anticancer efficacy on pediatric PBT24 cell line tumors were found in the literature. The pediatric U373 and U373vIII cell line GB tumors, formed by being intracranially injected into female athymic nude mice, are sensitive to NaDCA treatment, and it decreased the pyruvate dehydrogenase kinase 1 expression in the tumors.^16^ NaDCA significantly inhibited several pediatric high-grade gliomas cells’ viability.^17^

The pharmacological effect of the difference between NaDCA and MgDCA in cancer treatment was not previously studied. Differences between the effect of NaDCA and MgDCA on tumor growth indicate that the impact of dichloroacetate depends on interference with the Na^+^ or Mg^2+^ effect; on the other hand, these characteristics may indirectly reflect some variations in U87 MG and PBT24 cells biology.

NaDCA decreases NKCC1 expression in rat thymocytes.^20^ GB cell accumulates [Cl^-^]_i_ to levels ∼10-fold higher than average.^44^ GB cell [Cl^−^]i homeostasis disruption is related to upregulated NKCC1.^45^ A potential NKCC1 role is a participation in cell proliferation in tumors with high NKCC1 expression, such as glioma.^46^ In human glioma, NKCC1 protein expression positively correlates with tumor grade.^44^ Pharmacological inhibition of NKCC1 reduces glioma cell migration and invasion.^44,47^ Differences in the effect of 10 mM and 5 mM NaDCA on U87 MG and PBT24 cells may be due to the force of Na^+^ ions. Increased Na^+^ concentration may induce activation of NKCC1 as well as cause metabolic changes in U87 MG and PBT24 tumor cells differently. Increased extracellular Na^+^ concentration can stimulate cell NKCC1 activity.^48^ That would be consistent with the Na^+^ replacement with Mg^2+^ in the dichloroacetate salt; MgDCA acts equally on U87 and PBT24 tumor growth. Na^+^ and Cl^-^ ions in a tumor microenvironment are involved in cancer progression.^26,46^

This study indicates that an additional criterion for assessing malignancy of xenograft is the thickening of the CAM, and these changes are directly related to an increased number of blood vessels in CAM mesenchyme. The non-treated U87 and PBT24 xenografts induced the CAM thickening similarly. Others reported that xenograft induces an inflammatory response in the CAM region.^49-51^ The PBS solution used as a control does not activate, but hyperosmolar solutions enable membrane thickening.^52^ CAM membrane thickening is inseparable from neoangiogenesis.^53^ A tumor on CAM induces angiogenesis with the increased thickness of membrane mesenchyme.^49,52^ The vessel plexus was manifested around the xenograft after 5 days of U87 or PBT24 tumor grafted on CAM. The CAM thickness was insignificantly increased in the U87 tumor that was treated with 10 mM of NaDCA compared to the control. The opposite effect of 10 mM NaDCA was found in the PBT24 tumor compared to the control, with a suppression of the CAM thickness and vascular number. Others reported that NaDCA therapy suppressed angiogenesis *in vivo.* ^19^ This study shows that the effect of 10 mM dichloroacetate anion concentration on CAM thickening is dependent on the cation in the preparation (10 mM NaDCA and 5 mM MgDCA).

Patients with GB are defined by hypomagnesemia.^54^ Mg^2+^ is required in mitochondrial function and glycolysis processes.^55^ The relevance of intracellular and extracellular Mg^2+^ concentration to tumorigenesis has been shown to be associated with contradictory data.^56,57^ Elucidation of the relationship between Mg^2+^ and tumorigenesis by preclinical data could be significant in the clarification of Mg^2+^ homeostasis disorders.^56^

This study shows that the PCNA-positive cells percentage in U87 and PBT24 control tumors were comparable. PCNA is a nuclear marker of cell proliferation occurring only in proliferating cells, and its expression increases with GB grade.^58^ Both 5 mM of NaDCA and 5 mM of MgDCA treatment decreased PCNA expression in U87 and PBT24 tumors similarly. Researchers have reported that PCNA in the glioma of adult patients showed PCNA as an independent prognostic indicator, and its increased expression correlated with decreased patient survival.^58^ Pediatric and adult metastatic or relapsed high-grade gliomas have a higher PCNA appearance.^58,59^ Its expression permits evaluation of the efficacy of cancer treatment in a CAM model.^60^

A CAM model is relevant in testing investigational medicine designed to interfere with EZH2 molecular pathways in cancer.^28,61^ EZH2 inhibits genes accountable for suppressing tumorigenesis, and inhibiting EZH2 activity may reduce tumor growth.^62^ This study shows that EZH2 expression in U87 and PBT24 control tumors were similar. The treatment with NaDCA, as well as MgDCA preparation, significantly decreased EZH2 appearance in the U87 and PBT24 tumors. The EZH2 appearance suppression was highest in the U87 tumor treated with 5 mM of NaDCA and 5 mM of MgDCA, sequentially 8.3- and 20.5-times, while in the PBT24 tumor the preparations reduced EZH2 expression by 3.8- and 4.0-times, respectively. This would mean that the cancer of the adult U87 cell line in terms of EZH2 appearance is more sensitive to the treatment with MgDCA than the pediatric PBT24 cell line tumor. A meta-analysis of 6 studies showed that EZH2 overexpression is associated with poor prognosis of high-grade pediatric and adult glioma.^63^ EZH2 is an anticancer drug target.^64^

Furthermore, preclinical studies show a synergistic effect of NaDCA with the chemotherapy applied in various cancers.^24^ The preclinical therapeutic approach of NaDCA combination with other medications suggested it could be incorporated into clinical trials.^19,36,42^ The risk of peripheral neuropathy restricts the treatment with NaDCA in adults. This adverse drug reaction is age-dependent, and the NaDCA dose can be escalated in pediatric patients without significant side effects.^17,65^

The targeting of tumor mitochondrial metabolism is a potential cancer treatment strategy. The U87 MG and PBT24 cell line tumors study shows the effect the U87 and PBT24 tumor differences have on the impact of varying NaDCA concentrations on tumor growth, and on the PCNA and EZH2 expression in the tumor cell. The variations between NaDCA and MgDCA efficacy on tumorigenesis may reflect differences in some U87 MG and PBT24 cell biology.

## Conclusion

The human glioblastoma U87 MG and PBT24 cell line tumors response variations to treatment with different sodium dichloroacetate concentration on tumor growth or a contrast between sodium dichloroacetate and magnesium dichloroacetate effectiveness may reflect some differences in U87 MG and PBT24 cell biology.
